# Methods for Establishing a Rat Model of Rheumatic Heart Disease

**DOI:** 10.31083/j.rcm2509346

**Published:** 2024-09-24

**Authors:** Shenglin Xian, Zhiyu Zeng

**Affiliations:** ^1^Department of Cardiology, The First Affiliated Hospital of Guangxi Medical University, 530021 Nanning, Guangxi, China; ^2^Guangxi Key Laboratory of Precision Medicine in Cardio-cerebrovascular Diseases Control and Prevention, 530021 Nanning, Guangxi, China; ^3^Guangxi Clinical Research Center for Cardio-cerebrovascular Diseases, 530021 Nanning, Guangxi, China

**Keywords:** rheumatic heart disease, rat model, method

## Abstract

Rheumatic heart disease (RHD) is responsible for nearly 250,000 deaths annually and poses a significant health threat in developing areas. The unclear pathogenesis of RHD makes the development of cost-effective treatments challenging, particularly as current surgical options are expensive and technologically demanding, exacerbating the economic and quality-of-life burdens for patients. Given the risks associated with direct human experimentation due to the uncertain pathogenesis, using a rat model infected with Group A Streptococcus (GAS) has become a crucial experimental strategy for RHD research. The development of an RHD rat model, refined over 23 years, now stands as a pivotal approach in studies aiming to understand the disease’s pathogenesis. This review summarizes the evolution, characteristics, advantages, and limitations of the RHD rat model, offering insights into potential areas for improvement. It aims to provide researchers with a comprehensive understanding of the model, supporting the advancement of research methodologies and the discovery of innovative treatments for RHD.

## 1. Background

Rheumatic heart disease (RHD) primarily affects young patients in developing regions, presenting a serious threat to public health, with approximately 250,000 annual fatalities [1, 2]. The pathogenesis of RHD remains poorly understood, complicating efforts to develop effective prevention and treatment strategies [3]. Surgery is currently the main treatment, though it requires long-term medication post-operation, is costly, and demands high technical expertise [4]. These factors make surgical intervention a significant burden for RHD patients in resource-limited settings, severely impacting their quality of life. The absence of preventive measures and effective drug treatments underscores the difficulties in the prevention and treatment of RHD. Understanding the pathogenesis of RHD is expected to fill these two major gaps.

Given the risks associated with direct human experimentation due to the unclear pathogenesis of RHD, animal models currently provide a better option for research. The human valve is similar in structure to the rat valve [5]. The human heart valve is made up of two layers of endothelial cells tightly covering connective tissue [6], endothelial and endothelial mesenchymal transformation markers were also detected on rat heart valves [7]. Echocardiography (ECG) techniques used in humans are applicable to rats, allowing for consistent assessment of heart anatomy, function, and hemodynamics [8]. The development of a rat model using Group A Streptococcus (GAS) to induce autoimmunity has become a pivotal advancement in RHD research [9]. This modelling method, first reported by Quinn *et al*. [9] in 2001, utilizes a recombinant streptococcal M protein capable of inducing valvulitis in Lewis rats. Building from this core methodology of engaging the body’s immune response to *Streptococcus* through antigen injection, a rat model of GAS induced autoimmunity can be successfully established [9]. This method has undergone many evolutions and developments in recent years, and it continues to help researchers establish reliable RHD rat models to study the pathogenesis of RHD.

This review summarizes the development and evolution of the RHD rat model method currently in use. We summarize the characteristics, advantages and disadvantages of this modelling method and discuss the space for improvement. This review will help to explore the pathogenesis of RHD and the development of the RHD rat model and provide a reference for researchers who use rat models to study RHD in the future.

## 2. Research Using the RHD Rat Model

In 1990, Kodama *et al*. [10] pioneered a rat model of myocarditis, selecting Lewis rats for their robust immune response. The core method for inducing immune response involved two key stages: initial and booster injections [10]. The initial injection consisted of equal volumes of antigen and complete Freund’s adjuvant (CFA), and was administered into the hind footpad [10]. To enhance the T helper 1 (Th1) immune response and promote autoimmunity, was the *Bordetella pertussis* vaccine [11] administered intravenously on the first and third days after the initial injection. The booster injection, mirroring the initial protocol, was administered on the 7th day. The experimental timeline ended on the 21st day after the initial injection, at which point the rats were sacrificed for analysis, completing a 21 day modelling period.

Although Kodama *et al*.’s method [10] is describing the establishment of a rat model of myocarditis, it has become the basis of subsequent methods for establishing a rat model of RHD because it uses the same principle of immune response as RHD modelling. Subsequent methods for establishing an RHD rat model, which will be discussed later in this review, were modified or improved on the basis of the methods of Kodama *et al*. [10]. Many aspects of their methods are still in use today, including the staged injection process (initial and booster), the use of the footpad as the injection site, and the use of CFA and inactivated *Bordetella pertussis* as adjuvant and coadjuvant, respectively [10], and initial injection of an equal-volume mixed solution of antigen and CFA. These elements are crucial for replicating the immune response necessary for RHD modeling, as will be further described in later sections of this review.

In 2001, Quinn *et al*. [9] pioneered the induction of RHD valvulitis in Lewis rats using recombinant type 6 streptococcal M protein (rM6). Their methodology, now considered classical, involved a two-stage induction process: an initial injection followed by a booster [9]. The rM6 antigen, combined with CFA as the main adjuvant and *Bordetella pertussis* cells as an additional adjuvant, was used to generate the RHD rat model [9].

### 2.1 Use of the Classical RHD Rat Model

In 2002, Galvin *et al*. [12] refined the RHD rat model based on the methods of Quinn *et al*. [9], focusing on myocarditis and valvulitis. They introduced cardiac myosin as an antigen to induce immune responses in 6–8 week old female Lewis rats [12]. The protocol once again utilized initial and booster injections. The initial injection site was the rat’s hindfootpad, and the injection solution was a 1:1 volume-to-volume(v/v) mixture of 0.5 mg of antigen and CFA. Anesthesia was provided with a short-acting solution of 10 mg of ketamine/0.2 mg of xylazine per rat before injection, minimizing adverse effects [12]. Additionally, inactivated *Bordetella pertussis* was used as a coadjuvant on Days 1 and 3 following the initial injection, with the booster injection mirroring the initial conditions, leading the study to conclude at 21 days after the initial injection [12]. The rats exhibited clinical symptoms of valvulitis and myocarditis, which was confirmed through hematoxylin and eosin (H&E) staining, specifically revealing autoimmune valvulitis [12]. The histopathological examination revealed valvular inflammation characterized by mononuclear cell infiltration [12]. Thus, substituting cardiac myosin as an antigen can also lead to a reproduction of clinical symptoms, which was validated through histological testing, demonstrating its efficacy in studying RHD. The modelling period was 21 days, which is not long and can be easily implemented.

In 2003, Lymbury *et al*. [13] reported that a mixture of peptides from the GAS M5 protein could induce valvulitis in Lewis rats. The animals used were still female Lewis rats aged 10 weeks. The modelling method consistent with that of Galvin *et al*. [12]. Their findings, based on lymphocyte proliferation assays and H&E staining, showed enhanced lymphocyte responsiveness and increased inflammatory cell presence in the cardiac valves [13]. Notably, there was significant monocyte infiltration within the valves and diffuse macrophage infiltration around the valve tissue [13]. Gorton *et al*. [14] established a rat model employed the same model to show cardiac lesions and histological evidence of either valvular or myocardial infiltration by inflammatory cells, along with elevated of CD4+ T-cell expression.

In conclusion, the rat model of RHD, originating from Kodama *et al*. [10], has been extensively validated through ELISA, lymphocyte proliferation assays, histology and immunohistochemistry. These techniques have consistently demonstrated the model’s ability to replicate RHD pathology effectively. This classical method is recognized for its efficacy, minimal harm to subjects, and short duration, making it a standard approach in RHD research.

### 2.2 Adjustment of the Modelling Period

In 2010, Xie *et al*. [15] deviated from the commonly used GAS M5 protein and instead utilized formalin-inactivated and then sonic-disrupted GAS to establish a rat rheumatic valvulitis model. The animals used were still female Lewis rats aged 7 weeks. The modelling process consisted of two stages: initial injection and booster injection. The solution for the initial injection was 0.2 mL of inactivated GAS and CFA mixed at a 1:1 ratio (v/v). The injection site was the hindfootpad. The booster injection was divided into two substages. In the first substage, 0.5 mL of inactivated GAS and CFA were subcutaneously injected at a 1:1 (v/v) ratio on the 7th and 14th days after the initial injection. In the second substage, 0.5 mL of inactivated GAS was subcutaneously injected every 7 days. The modelling process was terminated at 12 and 24 weeks after the initial injection. The method of anaesthesia before sacrificing the rats was intraperitoneal injection of 5% chloral hydrate. Then, histological examination was carried out, and Aschoff bodies, a characteristic of rheumatic carditis, were observed in the rat hearts after a modelling period of 24 weeks, indicating that this modelling period is suitable for establishing a chronic RHD rat model. At the same time, valvular disease (infiltration of lymphocytes, macrophages, and multinuclear giant cells, valve thickening, swelling, verrucous vegetation at the closed edge of the valve, and angiogenesis) was also observed. This study directly used inactivated GAS as an antigen and extended the modelling period from 21 days to 12 and 24 weeks, during which the RHD rat model was successfully established. The authors found that the 24-week modelling period is very suitable for the study of chronic RHD, which provides a good reference for future animal experimental research on chronic RHD.

In 2017, Xiao *et al*. [16] refined the classical RHD rat model by extending the time between the final booster injection and sacrifice to better observe disease progression. The authors found that acute RHD symptoms appeared two months after the final booster injection (Day 81), while chronic RHD symptoms appeared after four months after the final booster injection (Day 141) [16]. This adjustment in the modelling period demonstrated that extending the timeline allows for the clear distinction between acute and chronic RHD stages, providing a more effective framework for studying the disease’s long-term progression.

Therefore, both studies adjusted the 21-day modelling period of the classical modelling method and demonstrated that an extended modelling period can be used to establish an RHD rat model and that a chronic RHD rat model can be established after the modelling period is appropriately extended.

### 2.3 Footpad vs. Hock

In 2010, Gorton *et al*. [17] proposed a new injection site option, the hock, rather than the footpad. They determined that the hock injection could reduce adverse inflammatory effects and discomfort associated with the traditional footpad method [17]. Due to the adverse inflammatory side effects of CFA as an adjuvant, they tried to find suitable alternatives for CFA; however, they found that there was still no suitable adjuvant available to replace CFA. Finally, the effects of footpad injection and hock injection in the establishment of the RHD rat model were compared, as was the damage to the rats. It was found that hock injection can produce an immune response similar to that of footpad injection (valvulitis due to infiltration of CD4+ T helper cells), can also effectively alleviate the adverse inflammatory side effects caused by CFA, and can reduce pain, pressure and the possibility of infection in rats. The use of the hock site was validated in subsequent studies by Gorton *et al*. [18] in 2016, which also confirmed the method’s effectiveness through various assessments such as ECG, ELISA, lymphocyte proliferation assays, and histology. The result of ECG showed that the P-R interval of the rats in RHD group was significantly prolonged compared to the rats in control group [18]. Other tests validated clinical characteristics of RHD including the appearance of valvulitis, myocarditis, and increased inflammatory cell infiltration with each subsequent rM5 booster injection [18]. These findings demonstrated that the modelling method using the hock as the injection site can also be successful. Similarly, in 2016, Batzloff *et al*. [19] once again used the hock injection method and noted significant reductions in inflammation and arthritis symptoms in the rats, with histology confirming successful induction of cardiac valvulitis.

The methodological shift to using the hock for injections not only enhanced the welfare of the experimental rats but also preserved the validity of the RHD model. The reduction of inflammation and arthritis in the hindfoot is particularly crucial for Lewis rats, which are susceptible autoimmune diseases and may experience adverse effects on their eating habits, activity levels, and overall well-being [20, 21]. The successful implementation of this approach by Gorton *et al*. [17, 18] highlights potential avenues for further refining the classical RHD rat model. Moreover, it underscores the ethical imperative in animal research to minimize discomfort and pain for experimental subjects.

### 2.4 Adjustment of the Injection Solution

#### 2.4.1 Inactivated GAS

Building on the 2010 experiments by Xie *et al*. [15] which successfully used sonicated and inactivated GAS as the injection solution for an RHD rat model, Wen *et al*. [22] in 2015 updated these methods to further explore Th17-related cytokine changes in RHD. Instead of the M5 protein, the authors utilized the entire GAS organism, inactivated and sonicated [22]. The adjuvant remained CFA, and the injection protocol was consistent with the earlier study [22]. Post-modeling analyses, including H&E staining, revealed the presence of Anitschkow cells (ACs), loose edema, inflammatory exudation, and focal lymphocyte infiltration of valve tissue [22]. It is known that ACs are stromal cells which can be found in the heart muscle, heart valves and coronary vessel walls [23]. When the human body suffers from rheumatic fever, ACs can be observed in the human heart and serve as histological signs of Aschoff nodules [23]. This study proved that using inactivated GAS as an antigen can not only lead to the successful establishment of an RHD rat model but also greatly simplify the antigen preparation process.

#### 2.4.2 Group G Streptococcus (GGS)

In 2018, Sikder *et al*. [24] explored the use of GGS alongside GAS as antigens to establish an RHD rat model. Employing the same protocol for both antigens, they utilized hock injections and differentiated the booster stage into short- and long-term injections [24]. The booster regimen included three short-term injections administered once every 7 days and five long-term injections on Days 7, 14, 21, 120, and 210 after the initial injection [24]. The effectiveness of both antigens was compared using ECG, histological, and ELISA analyses, which demonstrated similar immune responses induced by GGS and GAS. This study not only validated GGS as a potential antigen for modeling RHD but also suggested that various streptococcal groups might be involved in the disease’s pathogenesis.

#### 2.4.3 Effector T Cells Generated by the GAS M Protein

In 2019, Sikder *et al*. [25] investigated the potential of using antibodies and effector T cells generated from GAS M protein injections to establish an RHD rat model. Lewis rats were categorized into donor and recipient groups [25]. Donor rats received injections of GAS M protein according to the classical RHD model and subsequently, their serum (containing anti-GAS M5 antibodies) and spleen cells (including T cells) were injected into recipient rats [25]. Evaluations through ECG, histological, and ELISA analyses demonstrated that these antibodies and effector T cells could enter heart tissue and cross-react with its proteins, thereby inducing an autoimmune response [25]. This study highlighted a novel approach to model RHD and underscored the significant role of effector T cells in mediating autoimmune responses post-GAS infection, offering insights into the disease’s pathogenesis.

#### 2.4.4 Cardiac Myosin

As mentioned above, in 2002 Galvin *et al*. [12] used cardiac myosin as an antigen to generate an RHD rat model. While various substances, including cardiac myosin, have been used as antigens to establish RHD models, a direct or side-by-side comparison assessing their safety, immune efficacy, effects on other organs, risks to researchers, and operational ease has not yet been conducted. Such comparative studies are crucial as they can identify the most effective antigens and contribute significantly to understanding the poorly understood pathogenesis of RHD. Researchers are encouraged to design studies that focus on these comparative analyses to enhance the development of RHD models and advance our knowledge of the disease.

### 2.5 Adjustment of the Modelling Process

In 2019, Wu *et al*. [3] and Chen *et al*. [26] built on the methods established by Wen *et al*. [22] in 2015 to create RHD rat models, primarily modifying the booster injection stage, to study RHD-related signaling pathways [27]. The modified booster stage was divided into two substages: the first involved four weekly injections of 0.5 mL of inactivated GAS mixed with CFA were injected at a ratio of 1:1 (v/v), and the second involved four weekly injections of 0.5 mL of inactivated GAS alone [3, 26, 27]. The total modelling period extended to 63 days [3, 26, 27]. Assessments included Sirius red staining, which revealed significantly greater valve fibrosis in the RHD rats compared to the control group, and polymerase chain reaction (PCR) analysis that showed elevated expression of fibrosis-related molecular markers compared to the control group [3, 26, 27]. Moreover, immunohistochemistry also indicated increases in inflammatory factor levels near the valves [3, 26, 27]. These adjustments demonstrate that altering the booster injection schedule in the classical modeling method can effectively establish a successful RHD rat model.

### 2.6 Symptoms of Neurobehavioral Changes in RHD Rat Model

In 2023, Reynolds *et al*. [28] detected neurobehavioral changes in an RHD rat model, specifically symptoms of Sydenham’s chorea (SC), a neurological disorder following streptococcal infection. The behavioral tests include fine motor control (manipulation of food), gait and balance (beam walking), compulsive behavior (grooming and marble burying) and anxiety like behavior (light-box and dark-box tests) [28]. They findings revealed that RHD rats showed significant changes in food manipulation, grooming, marble burying behaviors, and experienced a prolonged time crossing the beam compared to control rats [28]. These results underscore SC as a crucial neurobehavioral consequence of streptococcal infection. In future research, SC can serve as vital indicators for observing the impact of streptococcal infection on the nervous system in RHD.

### 2.7 Using RHD Model Rats to Study the Safety of Candidate Vaccines

Both Batzloff *et al*. [19] and Reynolds *et al*. [28], as mentioned earlier, assessed the safety of candidate vaccines in RHD rat models. They focused on J8 (derived from a cryptic epitope in the C3 repeat sequence of GAS M protein), p*17 (from GAS M-protein epitopes), and K4S2 (from the interleukin-8 (IL-8) degrading *S. pyogenes* Cell-Envelope Proteinase [SpyCEP] epitope) [19, 28]. Each vaccine was formulated with diphtheria toxoid (DT) as the carrier protein and administered by subcutaneous injection at a dosage of 150 μg mixed with CFA 1:1 (v/v) [19, 28]. Safety evaluations showed that all three candidate vaccines were effective in treating RHD, and did not produce antibodies that cross react with human cardiac proteins or induce cardiac tissue inflammation, confirming their safety [19, 28]. These studies highlight the crucial role of the RHD rat model in advancing vaccine safety and therapeutic research for RHD, providing valuable insights for clinical applications.

### 2.8 The Downsides of Comparisons with Histological Pathological Studies of Rheumatic Carditis

The pathogenesis of RHD remains poorly understood, with valvulitis identified as the most common lesion in in histopathological studies [29]. The RHD rat models, established using the methods discussed in this review, show pathological similarities with human RHD cases, highlighting their utility despite the ongoing uncertainty about the disease’s pathogenesis and limitations in classical pathology descriptions.

In the context of this unclear pathogenesis, direct intervention experiments on RHD patients are nearly impossible. Currently, researchers can only analyze valve tissue from patients, either obtained post-mortem or during valve replacement surgeries. This typically results in studies based on post-mortem examinations of patients who succumbed to acute heart attacks, with small sample sizes potentially skewing our understanding of the disease. Ideally, valve tissue from patients undergoing valve replacement surgery would serve as a better experimental group. However, obtaining normal heart valves for control groups is challenging. An alternative is using valve tissue from surgeries for conditions like valve prolapse or degeneration, though these samples may not accurately reflect healthy tissue [22].

The RHD rat model allows for controlled interventions, and remains a critical tool for studying RHD. The primary criterion for evaluating the success of these models is the pathological similarity between the valve tissues of the rats and human patients. This standard, however, is also constrained by the incomplete understanding of RHD’s pathogenesis. Despite these limitations, the model’s relevance is reinforced by ongoing efforts to refine it, including adjustments to injection sites and antigens by researchers like Gorton *et al*. [17, 18] and Xie *et al*. [15]. Further innovations include exploring neurobehavioral symptoms by Reynolds *et al*. [28] and performing ECG analysis on the Gorton *et al*. [18] model.

As researchers continue to enhance the modeling process and expand the scope of their studies, the RHD rat model stands as the most reliable method for mimicking the pathological characteristics of human RHD, even as it faces inherent challenges. This ongoing improvement underscores the model’s potential to deepen our understanding of RHD, pointing to areas for future research and methodological enhancements.

## 3. Discussion

In all the studies mentioned above, Lewis rats were used as experimental animals to establish the RHD models. These rats are highly susceptible to developing autoimmune diseases [20, 21], which aligns with the widely recognized theory that RHD pathogenesis involves autoimmunity [30]. Due to their high susceptibility, Lewis rats are more prone to developing RHD, making it easier to establish RHD models with them compared to ordinary rats.

### 3.1 Adjustment of the RHD Rat Model

In the evolution of the RHD rat model, researchers have been able to customize their approach to suit specific conditions and experimental objectives. Experimentally validated adjustments include the type of injection—ranging from GAS M protein, GAS, GGS, to effector T cells generated by the GAS M protein [9, 13, 24]. Choices of injection site have been expanded from the traditional footpad to include the hock, which has been found to be less harmful [17, 18, 19]. Anesthesia methods vary, including intraperitoneal anesthesia with ketamine and xylazine, isoflurane in 100% oxygen, or a combination of 5% isoflurane inhalant with 1–2 L/min O_2_ delivered via a nose cone, with 2% isoflurane maintained during procedures [12, 19, 24]. The length of the modeling period also offers options from as brief as 21 days to as extended as 210 days, which can influence whether acute or chronic RHD symptoms develop [15, 16]. Furthermore, the modeling process itself may consist of an initial injection followed by a booster injection, or an initial injection followed by two subdivided stages of booster injections [3, 26]. These modifications allow for a flexible yet precise approach to modeling RHD in rats, facilitating tailored investigations into the disease.

The RHD model is considered safe for researchers as it does not provoke aggressive behavior in the rats. However, the modelling process involves numerous injections, necessitating careful handling to prevent accidental injuries from the syringe needles. Establishing the RHD rat model does not require highly specialized experimental conditions, making it accessible for many laboratories. One notable consideration is the potential damage from footpad injections, as the hind feet are essential for the rats’ support during eating and drinking. To mitigate this, researchers should implement protective measures, such as placing soft cushions at the bottom of the cages. Alternatively, reducing the injection volume in the footpad can help minimize adverse effects while ensuring successful modeling. A brief description of the method used for establishing the RHD rat model is provided in Table 1.

**Table 1.** **Overview of Methodological Variations in Establishing RHD Rat Models (2001–2024)**.

Parameter	Details
Time	2001–2024
Animal	Lewis rat
Sex	Female
Age (week)	8–14
Antigen	1. GAS M protein; 2. Cardiac myosin; 3. GAS; 4. GGS; 5. Effector T-cells generated by GAS M protein
Coadjuvant	Complete Freund’s adjuvant
Modelling period	21 days; 63 days; 81 days; 141 days; 84 days; 168 days; 210 days
Modelling stages	1. Initial injection and booster injection; 2. Initial injection and two subdivided stages of booster injection
Anaesthesia method	1. Intraperitoneal anesthetization with ketamine and xylazine at 10 mg and 0.2 mg per 250 g of body weight respectively; 2. Anaesthesia with isoflurane (5%) in 100% oxygen; 3. Nose cone with 5% isoflurane inhalant and 1–2 L/min O_2_ in a gaseous anaesthetic machine with 2% isoflurane for maintenance of anaesthesia during interventions
Solution for the initial injection	1:1 (v/v) mixed solution of antigen and CFA
Site for the initial injection	Rat hind footpad or hock
Solution for the booster injection	Same as the initial injection solution or without CFA
Site for the booster injection	Abdominal subcutaneous
Number and interval of injections	One injection weekly
Method of sampling	Cardiac tissue was collected and blood was collected from tail vein or fundus vein
Sample	Cardiac tissue and blood

This table summarizes the variations in methodology for establishing RHD rat models over the period from 2001 to 2024. The parameters listed include the timeline of studies, the specific animal model used (Lewis rat), and key experimental conditions such as sex, age range, types of antigens, injection methodology and sampling. RHD, rheumatic heart disease; GAS, Group A Streptococcus; GGS, Group G Streptococcus; CFA, complete Freund’s adjuvant; v/v, volume/volume.

In addition to the existing adjustments, further modifications may be explored in the future to enhance the RHD rat model. One area of improvement involves the injection volume. The current method involves injecting 0.5 mL of solution subcutaneously after the initial injection, which can cause severe pain, irritation, and local inflammation [17]. Future studies could focus on establishing a gradient of injection volumes to determine the minimum volume necessary to successfully model RHD while minimizing discomfort. Another potential adjustment involves the method of inducing an immune response. All the existing studies induce immunity through injection. Considering that GAS can also infect through the respiratory tract, exploring respiratory infection methods such as nebulized inhalation could be viable. This approach may reduce the damage and stress associated with injections and anesthesia. However, this method increases the risk of infection, as well as the risk of contaminating the laboratory environment, necessitating meticulous operation, strict safety protocols, and specialized areas in the laboratory. Lastly, the selection of injection sites is currently limited to the footpad and the hock. Future studies could evaluate the effects of different injection sites on immune response induction. This could help identify more effective and safer injection sites for rats, further refining the model’s efficacy and safety.

### 3.2 New Ideas for Studying the Pathogenesis of RHD with RHD Rat Models

Despite the annual toll of 250,000 deaths [2] and significant impact on quality of life, the pathogenesis of RHD remains poorly understood, hindering effective prevention and treatment. Current research primarily employs RHD rat models to explore activated signaling pathways or to compare samples from RHD patients to controls to achieve similar insights [3, 31, 32, 33, 34, 35]. The establishment of an RHD rat model is critical as it forms the foundation for further studies into the disease’s pathogenesis. Future research could shift focus from using the successful establishment of RHD models as a starting point to making it the endpoint. This would involve adjusting each step of the modeling process to see if rats can still successfully develop RHD, thus helping identify which stages directly influence pathogenesis.

In addition, exploring different antigens that induce RHD in rats could provide deeper insights into the disease’s mechanisms. Previous studies all used female rats, incorporating both sexes could be used to determine the relationship between sex and RHD. Moreover, the organs targeted by previous studies included almost all valves and the myocardium. It is possible that different organs of rats could be preserved to study the changes in different organs during the onset of RHD. Advanced techniques like proteomics or transcriptomics could elucidate the similarities and differences in biological pathways between the rat model and human acute rheumatic fever (ARF). For instance, the persistence of heart reactive antibodies (HRA)-immunoglobulin G (IgG) [36] and recent findings on the IgG3-C4 reaction [37] in ARF highlight potential intervention targets. Utilizing the RHD rat model’s manipulability could significantly advance our understanding of the disease’s pathogenesis and inform therapeutic strategies.

## 4. Conclusions

We have demonstrated that RHD poses a significant health threat with a pathogenesis that remains poorly understood. This uncertainty makes it difficult to conduct safe experimental studies directly on humans. However, the RHD rat model, in use for over 23 years, has proven to be an invaluable tool for studying the disease [9]. It offers a simple, safe, and highly successful approach that has continuously aided researchers in their exploration of RHD. The dedication of researchers to refining this model is commendable and warrants respect. Looking ahead, there are opportunities to enhance the model’s treatment efficacy and safety for the rats, particularly focusing on different aspects of RHD. Detailed methodological adjustments could increase the accuracy and success rates of experiments. Ongoing improvements to the RHD rat model are essential for advancing our understanding of the disease’s pathogenesis and developing effective prevention strategies.
